# Increasing the Dietary Concentration of *Lupinus albus* L. Decreased Feed Intake and Daily Gain of Immunocastrated Male Pigs

**DOI:** 10.3390/ani11071866

**Published:** 2021-06-23

**Authors:** Karen L. Moore, Emalyn Loudon, Frank R. Dunshea

**Affiliations:** 1 Pork Innovation WA Inc., Viveash, WA 3056, Australia; emalyn@live.com.au; 2 Faculty of Veterinary and Agricultural Sciences, The University of Melbourne, Parkville, VIC 3010, Australia; fdunshea@unimelb.edu.au; 3 Faculty of Biological Sciences, University of Leeds, Leeds LS2 9JT, UK

**Keywords:** albus lupins, immunocastrated male pigs, growth performance

## Abstract

**Simple Summary:**

Boar taint is a major cause of consumer complaints about pork but it can be eliminated by immunizing entire males against gonadotrohpin releasing factor (immunocastrated males). However, immunocastrated males have increased feed intake and backfat compared to entire males, which is an issue in countries such as Australia, where producers are penalized for high backfat. The inclusion of an in-feed ingredient, albus lupins, is one way to suppress the voluntary intake of IC male pigs and subsequently their fat deposition, but the appropriate quantity to include in the diet had not been determined. Albus lupins are thought to reduce feed intake due to slower transit of feed through the digestive system, which then influences satiety. The albus lupin concentration to maximize growth rate, minimize feed intake, maximize carcass weight and minimize backfat depth was 120, 142, 62.7 and 138 g/kg, respectively.

**Abstract:**

An experiment was conducted to determine the appropriate dietary concentration of albus lupins that would lower feed intake and decrease backfat while optimizing the effect on the growth rate of immunocastrated male pigs. The pigs were fed albus lupins (varying from 0 to 200 g/kg) from 2 weeks after the last immunization against GnRF for 14 d prior to slaughter (where d 0 is the day of the last immunization against GnRF). Increasing the dietary albus lupin concentration decreased daily gain for d 15 to 28 (*p* = 0.004). Daily feed intake also decreased as the concentration of the albus lupins increased for d 15 to 28 (*p* < 0.001). Carcass weight and backfat decreased as the concentration of dietary albus lupins increased (*p* = 0.011 and *p* = 0.024, respectively). The albus lupin concentration to maximize growth rate, minimize feed intake, maximize carcass weight and minimize backfat depth was 120, 142, 62.7 and 138 g/kg, respectively.

## 1. Introduction

Boar taint is an offensive smell that can occur when meat from entire males is cooked; however, the issue can be eliminated by immunizing entire males against gonadotrophin releasing factor (GnRF) [1,2]. Pigs immunized against GnRF (immunocastrated males) grow faster than entire males but consume more feed, resulting in high backfat deposition [3,4,5,6,7,8]. This is an issue in countries such as Australia, where producers receive a reduction in carcass price per kilogram from the processor for too much backfat.

One way to reduce the feed intake of immunocastrated pigs is to restrictively feed pigs through either quanitative or qualtitative restriction. Quantitative restriction occurs when the amount of feed fed is restricted, and several researchers have shown that immunocastrated males fed restrictively have less backfat [9] and reduced carcass fatness [10,11]. However, when pigs are group-housed, restricting the amount of feed can increase aggression [10]. Qualtitative feed restriction occurs when feed is offered ad libitum but ingredients are included that, for example, reduce feed quality or suppress appetite [12]. One ingredient known to reduce feed intake in pig diets is *Lupinus albus* L. (albus lupins) [13,14,15]. The mechanism by which albus lupins decreases feed intake is not entirely known but is thought to be due to slower transit of feed through the digestive system, which then influences satiety [13,15].

Previous studies investigated the inclusion of albus lupins in the diets of immunocastrated males to reduce feed intake and backfat [6,16]. It was found that the inclusion of albus lupins at 20% in the diet of immunocastrated males reduced feed intake, improved feed conversion and reduced backfat, but the impact on growth rate was inconsistent (reduced to either similar to or significantly lower than entire males fed a standard finisher diet) between the studies. Although including albus lupins in the diets of immunocastrated males has shown the potential to decrease both feed intake and backfat, the most appropriate inclusion concentration still needs to be determined due to the differences in findings in growth performance and feed intake between our studies [6,16].

It is desirable to identify the most appropriate concentration of albus lupins in the diet in order to maximize the decrease in feed intake and subsequently backfat but, at the same time, minimizing the negative effect on daily gain. If an appropriate amount of albus lupins can be determined, then the production of immunocastrated males may be optimized for producers. The objective of the study was to determine the concentration of albus lupins in the diet of immunocastraled males that would maximize the decrease in feed intake and backfat while optimizing the growth rate. The hypotheses were (1) as the albus lupin concentration in the diet of immunocastrated males increases, the feed intake and daily gain of the pigs will decrease until a plateau is reached at the optimum inclusion concentration and (2) the backfat of pigs immunized against GnRF will decrease as the albus lupin concentration in the diet increases.

## 2. Materials and Methods

### 2.1. Ethics Statement

The experiment was approved by the Department of Primary Industries and Regional Development’s Animal Ethics Committee (Activity number 17-6-13). The Australian code of practice for the care and use of animals for scientific purposes [17] was followed for the handling of the pigs.

### 2.2. Experimental Design, Animals and Housing

Six concentrations of albus lupins (variety Amira, 0, 40, 80, 120, 160 and 200 g/kg) were used in a completely randomized experiment. Two hundred and sixteen male pigs (Large White × Landrace × Duroc) who had received their priming dose of the anti-gonadotropin releasing factor vaccine (Improvac^®^, Zoetis Australia, Rhodes, Australia) were sourced at 72.6 ± 6.13 kg liveweight from a commercial facility. The pigs were randomly allocated to treatment after being ear-tagged and individually weighed. The pigs were group-housed (6 pigs per pen with 6 replicate pens per treatment) in a grower/finisher shed with natural ventilation. The pigs had ad libitum access to feed and water.

### 2.3. Feeding Regime and Diets

All pigs received their second dose of Improvac^®^ and were fed the same diet (diet d 0–14, Table 1) from d 0. From d 15, the pigs were fed their allocated experimental diets for 2 weeks pre-slaughter. These diets had a lower nutrient profile than the diet d 0-4 based on the findings of Moore et al. [18]. The experimental diets contained 0, 40, 80, 120, 160 or 200 g/kg albus lupins and had 13.5 MJ dietary energy (DE) and 0.50 g standardized ileal digestible lysine (SID)/MJ DE. The two extreme diets (0 g/kg albus lupins and 200 g/kg albus lupins) were blended using a Feedlogic system (automated feed delivery system, FeedPro, Feedlogic Corp., Wilmar, MN, USA) in the appropriate ratio to obtain the four middle diets. For example, to obtain the 80 g/kg albus lupins diet, 60% of the diet was the 0 g/kg diet and 40% of the diet was the 200 g/kg diet. The quantitative amino acid composition (Australian Proteome Analysis Facility, Sydney, NSW, Australia) of the diets is given in Table 2.

### 2.4. Growth Performance

Pigs were weighed on d 0, 14 and 28. Feed intake was determined on a pen basis by the amount of feed delivered by the feeding system to the feeder. Daily gain, feed intake and the feed conversion ratio (feed intake/daily gain) were determined on a pen basis for the periods d 0 to 14, d 15 to 28 and d 0 to 28.

### 2.5. Slaughter Procedure

Twenty-eight days after the second vaccination against GnRF, all pigs were individually tattooed and taken to a commercial abattoir. The following day, the pigs were stunned using a carbon dioxide, dip-lift stunner set at 85% CO_2_ for 1.8 min (Butina, Denmark). Hot carcass weight (AUSMEAT Trim 13; head off, hind trotters on, fore trotters off; AUS-MEAT Ltd., South Brisbane, Qld, Australia) was determined prior to chiller entry approximately 35 min post-exsanguination. The backfat depth was measured at the point of the last rib and 65 mm from the dorsal midline (PorkScan Pty Ltd., Canberra, Australia).

### 2.6. Statistics

One-way analysis of variance was performed with the GENSTAT 18 program (VSN International Ltd., Hemel Hempstead, UK) with albus lupin concentration as the treatment. Pen was used as the experimental unit for all parameters. If *p* < 0.05, then the means were statistically different, while *p* < 0.1 but > 0.05 was determined to be a trend. Differences between treatments were determined using Fisher’s protected least significant differences. A split line regression fitted to the treatment means was used to determine the optimal albus lupin concentration to include in the diet when the values between treatments were significantly different.

## 3. Results

There were differences in the analyzed amounts of amino acids between the 0 g/kg and 200 g/kg albus diets. Despite the differences in analyzed amounts of amino acids, all (except methionine) were above the estimated standardized ileal digestible amino acid requirements of pigs from 80 to 120 kg LW, as outlined in NRC [19]. Methionine was lower in both the 0 g/kg Albus and 200 g/kg Albus diets than estimated in NRC [19].

### 3.1. Growth Performance

As expected, daily gain and feed intake were not different between treatments for d 0 to 14 (all *p* > 0.05, Table 3). As the albus lupin concentration in the diet increased, daily gain decreased from d 15 to 28 and for the overall period (*p* = 0.004 and *p* = 0.03, respectively). The feed intake of the pigs decreased as the albus lupin concentration in the diet increased from d 15 to 28 and d 0 to 28 (*p* < 0.001, *p* = 0.003, respectively). The feed conversion ratio was not affected by albus lupins for all periods (*p* > 0.05). As the albus lupin concentration in the diet increased, the final liveweight decreased (*p* = 0.005).

### 3.2. Carcass Data and Predicted Albus Lupin Concentration

Carcass weight and backfat were reduced as the amount of albus lupins increased (*p* = 0.01 and *p* = 0.02, respectively; Table 4). There was no effect of albus lupin concentration on dressing percentage (*p* > 0.05).

Using split line regression analysis, the predicted amount of albus lupins to minimize feed intake, maximize growth rate, maximize carcass weight and minimize backfat depth was 142, 120, 62.7 and 138 g/kg, respectively.

## 4. Discussion

The hypothesis that as the albus lupin concentration in the diet of immunocastrated male pigs increases, growth rate and feed intake will decrease until a plateau is reached at the optimum inclusion concentration was supported. A split line regression analysis found that the optimum dietary albus lupin concentration to maximize the growth rate was 120 g/kg. To minimize feed intake, the concentration was 142 g/kg. Although an optimum inclusion of albus lupins was determined, there was also a linear relationship between growth rate and feed intake and the amount of albus lupins, with growth rate and feed intake decreasing as the amount of albus lupins increased. This suggests that the pigs can detect even a small quantity of albus lupins in their diet.

As expected, feed intake increased considerably from d 15 to 28, amounting to a 52% increase in the pigs consuming the diet without any albus lupins, which has been found previously [7,8,9,20,21,22]. The increase in feed intake in immunocastrated males is attributed to changes in behavior (less aggressive and sexual behavior) due to a decrease in testosterone and estradiol [8,23].

Even in pigs consuming the diet with the highest amount of albus lupins, there was a 32% increase in feed intake from d 15 to 28, indicating the removal of a potent inhibition of feed intake in entire males. The decrease in the growth rate and feed intake of pigs when albus lupins were used in the diet at 20% concurs with Moore et al. [6] and Moore et al. [16]. The inclusion of albus lupins in the diet at 30% has also resulted in reductions in feed intake of between 12% and 27% in the finishing period [6,7]. In contrast, Kelly et al. [24] fed 10% to 30% albus lupins (cultivar Ultra) to pigs from 58 to 102 kg and found that feed intake and daily gain were reduced only by the inclusion of 30% albus lupins. The differences between studies may be attributed to the length of time for which the albus lupins were fed, with a longer feeding time potentially resulting in the pigs acclimatizing to the albus lupins in the diet.

When pigs are fed diets with albus lupins, the lower feed intake is thought to be mainly due to feedback on satiety signals caused by a slower transit by albus lupins through the digestive system [13,16,25]. Van Nevel et al. [14] also suggest that the decrease in feed intake may be due to bitter saponins in albus lupins or an increase in volatile acid production in the hind gut. However, a review by van Barneveld [26] concluded that it is unlikely that the reduced feed intake in pigs fed albus lupins is due to saponins as there are low levels present.

This study was partly undertaken due to conflicting results found in the magnitude of the decrease in growth rate and feed intake when albus lupins were included at 20% in the diets of immunocastrated males for 2 weeks pre-slaughter in Moore et al. [6] and Moore et al. [16]. In the 2 weeks pre-slaughter, Moore et al. [6] found that feed intake was decreased by 20% compared to the diet without albus lupins, while, in Moore et al. [16], the reduction was approximately 25%. In the present study, including albus lupins at 20% decreased feed intake by 15% compared to diets with no albus lupins. The large decrease in feed intake in Moore et al. [16] was attributed to increased acceptability issues with the albus lupin diet, which given the results of the present study, may have been associated with issues in the diet manufacturing.

The hypothesis that the backfat of pigs immunized against GnRF would decrease as the concentration of albus lupins in the diet increases was supported. Backfat decreased as the albus lupin concentration increased. To minimize the backfat thickness, the predicted amount of albus lupins in the diet is 138 g/kg. This decreased backfat thickness by approximately 9% (~1.2 mm) compared to the diet with no albus lupins in the current experiment. These findings concur with several other researchers who found that when albus lupins were included in finisher diets, backfat was reduced [6,11,14,27]. When excessive backfat of immunocastrated males affects market price, the decrease in backfat in the present experiment supports the dietary inclusion of albus lupins.

As demonstrated in the current experiment, including albus lupins in the diets of immunocastrated males will avoid the large increase in fat deposition and feed intake associated with their production. It also means that the improved social and aggressive behaviors of immunocastrated males compared to entire males can be maximized by allowing them to be kept for at least four weeks after the second dose against GnRF [4,28]. There are also likely to be fewer carcass lesions at slaughter due to the decrease in aggression [20].

The inclusion of albus lupins in the diet at varying concentrations did not affect dressing percentage. This is supported by Moore et al. [16], who, when feeding immunocastrated males 200 g/kg of albus lupins, found no difference in the dressing percentage when compared to those fed 0 g/kg albus lupins for 14 d pre-slaughter. In contrast, when immunocastrated males were fed 200 g/kg albus lupins [16] or 200 to 300 g/kg albus lupins [6] for 28 d pre-slaughter, the dressing percentage was decreased by between 0.8% and 1.5% compared to those who did not receive albus lupins. King et al. [29] also found that the dressing percentage decreased when albus lupins were included in the diet at 350 g/kg. A regression analysis conducted by Kim et al. [15] when albus lupins were fed at concentrations up to 300 g/kg also found that the dressing percentage decreased between 0.7 and 1.4 percentage units for every 100 g/kg inclusion of albus lupins. The regression analysis was based on work by Van Nevel et al. [14], who fed the cultivar Lublanc between 43 and 102 kg, and King [27], who fed the cultivar Hamburg from 22 to 70 kg. The decrease in dressing percentage as the concentration of albus lupins increased was attributed to the growth of gut tissue as a consequence of the growth of bacteria in the gastrointestinal tract due to high levels of non-starch polysaccharides, which are present in lupin seeds [15]. The differences in change in dressing percentages between studies may be attributed to the duration of feeding of the albus lupins as, perhaps, in the 14-day period in the current study, the growth in gut tissue was unable to be realized.

Various cultivars of albus lupins have been used in studies investigating the use of albus lupins in finishing pig diets. Kelly et al. [24] fed the cultivar Ultra and found reduced feed intake and growth rates when included at 300 g/kg. However, they noted that the cultivar (Ultra) used contained quinolizidine alkaloids. Donovan et al. [30] found no effect on feed intake when the same cultivar was included at 190 g/kg. Since the early studies on lupins, low-alkaloid lupins have been bred that are suitable for animal feeding, with albus lupins now containing approximately 0.1 g/kg air-dry basis alkaloids [15]. Other cultivars used in pig studies have included Hamburg, Lublanc and Kiev [13,14,27,29]. The present study and our previous studies [6,16] used the cultivar Amira. All of the cultivars of albus lupins used have resulted in a reduction in feed intake to various extents.

It is important to ensure that the inclusion of alternative feed ingredients does not affect meat quality. Including albus lupins in diets and its subsequent effect on meat quality was not explored in this study; however, a previous study by Moore et al. [6] found that the inclusion of albus lupins at between 20% and 30% for four weeks pre-slaughter had no effect on objective meat quality, except for pH_45_ min, which was higher in meat from pigs fed albus lupins compared to the conventional diet. No other studies were found that looked at the effect of albus lupins on meat quality; however, the inclusion of *Lupinus angustifolius* in varying concentrations up to 350 g/kg in pig diets did not affect meat quality [31].

## 5. Conclusions

Including albus lupins for two weeks pre-slaughter in the diets of immunocastrated male pigs successfully decreased feed intake and backfat. However, growth rate also decreased as the concentration increased. The amount of albus lupins to minimize feed intake, maximize growth rate, maximize carcass weight and minimize backfat depth was 142, 120, 62.7 and 138 g/kg, respectively.

## Figures and Tables

**Table 1:** Diet composition.

Ingredients, g/kg, As-Fed Basis	Diet d 0–14	0 g/kg Albus ^4^	200 g/kg Albus
Barley	24.32	49.39	41.48
Wheat	45	30	29.89
Mill run	5	5	5
Lupins, angustifolius	20	10	0
Lupins, albus (variety Amira) ^5^	0	0	20
Meat meal	4.18	2.31	2.00
Tallow	0.63	1.95	0.44
L-Lysine HCL	0.32	0.24	0.07
Methionine	0.087	0.023	0
Threonine	0.039	0	0
Tryptophan	0.011	0.001	0
Limestone	0.031	0.589	0.619
Salt	0.2	0.25	0.2
Choline chloride, 60%	0.060	0.132	0.180
Phytase ^1^	0.02	0.02	0.02
Vitamin and minerals ^2^	0.1	0.1	0.1
Nutrient composition ^3^			
Digestible energy, MJ/kg	13.5	13.5	13.5
Crude protein, g/kg	17.9	14.4	17.4
Ca, g/kg	0.80	0.80	0.80
Total P, g/kg	0.74	0.67	0.68
Available P, g/kg	0.59	0.52	0.52
Na, g/kg	0.13	0.13	0.11
NDF, g/kg	15.6	16.6	16.4
ADF, g/kg	7.14	6.20	6.70
g SID Lys/MJ DE ^4^	0.64	0.50	0.50

^1^ Phytase from Phyzyme Danisco Australia Pty Ltd.; ^2^ Provided per kilogram of final diet: Vitamins: A 8000 IU, E 700 g, D3 500 IU, K 2 g, B1 2 g, B2 5 g, B6 2.5 g, B12 20 mg; 40 mg pantothentic acid, 25 g niacin, 100 mg biotin, 1 g folic acid, 43 g calcium pantothenatic, 0.5 g cobalt, 60 g iron, 40 g manganese, 100 g zinc, 10 g copper, 1 g iodine, 0.2 g selenium, and 70 g antioxidant; ^3^ Calculated composition; ^4^ SID: Standardized ileal digestible lysine/MJ digestible energy; ^4^ Ratios of 0 g/kg Albus:200 g/kg Albus were 100:0, 80:20, 60:40, 40:60, 20:80 or 0:100 for 0, 40, 80, 120, 160 and 200 g/kg of albus lupins, respectively; ^5^ Whole lupins seed which was then ground as per normal milling processes.

**Table 2:** Amino acid composition of the diets.

Amino Acid (g/kg As-Fed Basis)	Diet d 0–14	0 g/kg Albus ^1^	200 g/kg Albus
Histidine	3.9	3.2	3.7
Isoleucine	5.7	4.8	6.2
Leucine	10.7	9.0	11.1
Lysine	9.2	7.0	7.5
Methionine	2.4	1.8	1.4
Phenylalanine	6.7	5.9	6.8
Threonine	5.7	4.5	5.6
Valine	6.9	6.0	7.2

^1^ The 0 g/kg Albus and 200 g/kg Albus diets were blended at the ratios 100:0, 80:20, 60:40, 40:60, 20:80 or 0:100 for 0, 40, 80, 120, 160 and 200 g/kg of albus lupins, respectively.

**Table 3:** Growth performance of pigs fed increasing concentrations of albus lupins (*n* = 6/treatment).

	Albus Lupins (g/kg)							
	0	40	80	120	160	200	SEM	*p*-Value
								
Initial LW ^1^	78.9	79.2	79.6	78.7	78.5	78.4	0.551	0.65
Final LW	114 ^a^	114 ^a^	113 ^ab^	111 ^bc^	112 ^ab^	110 ^c^	0.819	0.005
Daily Gain (kg/d)								
d 0–14	1.18	1.25	1.24	1.24	1.22	1.20	0.029	0.49
d 15–28	1.33 ^a^	1.22 ^ab^	1.16 ^bc^	1.09 ^bc^	1.19 ^ab^	1.03 ^c^	0.051	0.004
d 0–28	1.26 ^a^	1.24 ^ab^	1.20 ^abc^	1.17 ^bc^	1.21 ^ab^	1.12 ^c^	0.030	0.03
Feed Intake (kg/d)								
d 0–14	2.65	2.61	2.70	2.67	2.71	2.61	0.053	0.72
d 15–28	4.04 ^a^	4.05 ^a^	3.74 ^ab^	3.62 ^b^	3.59 ^b^	3.45 ^b^	0.126	0.01
d 0–28	3.34	3.33	3.22	3.14	3.14	3.05	0.077	0.08
Feed Conversion Ratio (kg/kg)								
d 0–14	2.25	2.09	2.17	2.16	2.22	2.18	0.069	0.65
d 15–28	3.04	3.35	3.25	3.34	3.04	3.42	0.137	0.25
d 0–28	2.66	2.70	2.68	2.71	2.61	2.74	0.070	0.84

^1^ LW: liveweight; ^a,b,c^ Means within a row with different superscripts differ significantly (*p* < 0.05).

**Table 4:** Carcass data for pigs fed increasing concentrations of albus lupins from 78.9 to 112.4 kg liveweight (*n* = 6/treatment).

	Albus Lupins (g/kg)							
	0	40	80	120	160	200	SEM	*p*-Value
Carcass weight (kg)	74.8 ^a^	74.9 ^a^	74.8 ^a^	72.6 ^bc^	73.8 ^ab^	71.5 ^c^	0.738	0.01
Dressing %	65.5	65.8	66.2	65.2	65.7	65.2	0.378	0.46
Backfat (mm)	12.2 ^a^	11.6 ^ab^	11.8 ^ab^	11.0 ^bc^	11.7 ^ab^	10.2 ^c^	0.398	0.02

^a,b,c^ Means within a row with different superscripts differ significantly (*p* < 0.05).

## Data Availability

Data may be made available for research purposes upon request to the corresponding author.
